# Online education and improvement of caring behaviors of nurses in pediatric wards: a quasi-experimental study

**DOI:** 10.1186/s12912-023-01315-8

**Published:** 2023-05-08

**Authors:** Elnaz Monemi, Monirsadat Nematollahi

**Affiliations:** 1 MSc in neonatal intensive nursing, nursing research center, Kerman University of Medical Sciences, Kerman, Iran; 2 Department of Pediatric and Neonatal Intensive Care Nursing, Razi Faculty of Nursing and Midwifery, Kerman University of Medical Sciences, Kerman, Iran

**Keywords:** Virtual education, Pediatric nursing care, Caring behaviors, Nurses, Pediatric wards

## Abstract

**Background:**

Nurses working in pediatric wards should communicate with children well and have appropriate caring behaviors according to the age of their patients, so distance education is very suitable for them due to its availability. This study aimed to determine the effect of online education on the principles of pediatric nursing care on the caring behaviors of nurses working in pediatric wards.

**Methods:**

This interventional (quasi-experimental) study used a simple random method to select 70 nurses working in pediatric wards and pediatric intensive care units in Kerman. The nurses in the intervention group received online training in the sky room three days a week, while nurses in the control group received routine pediatric care. The study instruments were the demographic information questionnaire and the Caring behaviors Questionnaire, which were completed by two groups before and one month after the intervention. Data were analyzed with SPSS 25. The significance level was set at P < 0.05.

**Results:**

The independent samples t-test indicated no significant differences in the mean scores of care behaviors between the intervention (256.61 ± 5.16) and control groups (257.52 ± 3.99) before the intervention (P = 0.23) but indicated a significant difference in the mean scores of caring behaviors between the intervention (275.69 ± 6.52) and control groups (254.21 ± 3.15) after the intervention. Therefore, online education increased the score of caring behaviors in the intervention group.

**Conclusion:**

Distance education had an impact on the caring behaviors of nurses in pediatric wards and we recommend the use of e-learning to improve the caring behaviors and the quality of care of nurses.

## Introduction

Care is one of the important tasks nurses do to provide health services to patients. Professional nursing care should respond to all aspects of human needs to improve patients’ health [1–3]. Care consists of physical and psychosocial dimensions: the physical dimension addresses the clinical components and skills of care, while the psychosocial dimension meets the psychological and emotional needs of patients [4, 5]. Meeting the needs of patients is one of the characteristics of quality care. The results of a study indicated that nurses focused on the physical aspects of care [6], while another study found no significant difference in the physical and psycho-social aspects of care [7]. Holistic nursing care should pay attention to all aspects of the patient’s existence [8]. Cowin and Johnson believed in the important impact of a deep perception of the concept of care on the quality of services and practices provided by nurses [9].

The nursing theory is based on the perception of care and caring behaviors [10], including nurses’ practices, cognitions, feelings, thoughts, imaginations, and actions when providing care to patients. Therefore, nursing care behaviors support, promote and preserve human values [4] and they are a combination of nursing practices and attitudes that relieve pain and meet the potential and actual needs of patients. Nurses’ caring behaviors show their concern about patients’ conditions, which conveys the nurse’s professional competence to the patient. Caring behaviors of nurses depend on their knowledge, attitudes, and skills [1].

Although caring behaviors vary from one society to another [1], their perception is a fundamental step in correcting nurses’ inappropriate caring behaviors and improving the quality of nursing care. Since the 1980s, Western countries have focused on nursing care rather than nursing theories and consider it as the heart of nursing practice [11].

Pediatric nursing is one of the most important areas in providing healthcare services [12] because pediatric nurses require special knowledge, experience, and skills [13]. Therefore, nurses’ caring behaviors depend on their clinical competence and their effective communication with parents and children before and after discharge [13]. Training helps pediatric nurses to improve their caring behaviors, master scientific principles, and provide standard care services [14]; therefore, training pediatric nurses is one of the most important aspects when providing healthcare services to children and their families [12]. Also, Factors such as psychological distress, burnout, previous training, being role models, and emotional intelligence [15] are effective in the quality of care behaviors of nurses [16].

Although caring is viewed as the central focus of nursing. However, despite its fundamental place in the clinical setting, researchers have failed in reaching a common definition. This failure has led to eliciting nebulous interpretations of the concept often leading to perplexity and opposing views between patients and nurses [17].

Nurses’ inappropriate caring behaviors will lead to irreparable results, so they must update their theoretical and practical knowledge [18, 19]. The training of nurses makes them ready to meet the health needs of patients (1) and provides conscious caring behaviors, which increase the feeling of security in patients and reduce their anxiety [11].

Various educational tools are necessary for training depending on learners such as educational booklets, videotapes, and distance learning through the Internet and educational CDs [20].

Nurses face problems during face-to-face training because it is not cost-effective and nurses have different educational needs, shift work, and insufficient free time to participate in training courses; therefore, they need intensive courses with the least cost and most effectiveness [21, 22].

E-learning can meet part of the educational needs of nurses and healthcare workers and increase the quality and effectiveness of education in providing care [22]. E-learning is distance education based on technology [21] and includes a wide range of educational methods such as computer-based education, web-based education, virtual classrooms, and software. Ease of access to educational content in e-learning provides the possibility of individual, situational, cooperative, and self-directed learning approaches [23]. Online courses are useful for training nurses due to their accessibility, low cost, and high productivity, and they provide a wide amount of new and varied information [24]. Although, for a better effect of online learning, the readiness of learners and access to technology is also very important. Also, the attitude of learners toward online education is important to the effectiveness of education[25]. Additionally, Some studies used distance learning offline, but due to the close relationship between the learner and the teacher in online learning, the presence of the learner in the class and the possibility of implementing new teaching methods in online classes are also possible [26]. Studies have shown the positive effect of virtual education on the care abilities of nurses in neonatal wards and emergency departments [22, 27].

Considering the importance of nurses’ ability to perform appropriate care behaviors in pediatric wards, the effect of training on their behaviors, as well as the role of virtual education in the current nursing education system, this study aimed to determine the effect of online education on the principles of pediatric nursing care on the caring behaviors of pediatric nurses.

## Methods

### Study design and setting

This interventional (quasi-experimental) study was performed with a pretest-posttest design. Seventy nurses (35 members in each group) working in the pediatric intensive care units and pediatric wards in Kerman participated in this study from March to May 2021. There are 300 pediatric nurses with more than 200 beds for children in Kerman.

## Sampling

A simple random method was used to select 300 nurses working in the pediatric intensive care unit and pediatric wards. Following a pilot study with 80% power and a 95% confidence interval and using Koopak formula, the sample size was estimated to be 60 participants, but 70 parents were selected for each group due to the 10% dropout rate.

None of the nurses were excluded from the study and thus the final sample included 70 nurses who participated in all stages of the study. We divided them into control and intervention groups using simple random sampling and a table of random numbers. The inclusion criteria were nurses with at least a bachelor’s degree in nursing and 6 months of work experience in one of the pediatric wards. The exclusion criteria were failure to complete at least 10% of the questionnaire, participate in the online classroom for more than 4 h or watch electronic content on WhatsApp channel within the specified time (4 weeks).

I**ntervention group**.

After obtaining the necessary permits and providing some information about the objectives of the study, the researcher received written consent from the nurses and enrolled eligible nurses using the random sampling method. Before the intervention, the researcher made the participants in the interventional groups familiar with the sky room system and assigned a username and password to each of the nurses. Then, the nurses in the two groups completed the demographic information questionnaire and the Caring Assessment Questionnaire. The nurses in the intervention group participated in a virtual classroom on pediatric care in the sky room three days a week for four weeks. They also received recorded content on WhatsApp every evening till four weeks after the end of the intervention. The training package covered all topics on basic pediatric care, which were extracted from the latest pediatric nursing care textbooks (Table 1). Each participant could watch the instructional materials as many times as needed by a link sent on WhatsApp.

**Table 1:** The content of the training program

Sessions	Comntents	
The First session	Getting to know the characteristics of childhood age groups Infancy, infancy, childhood, school age and adolescence How to properly communicate with a child Safety in children	
The Second session	Pain and its management importance in pediatric nursing care Methods of pain control in children Family and sick child (family-centered care)	
The Third session	Implementation of common procedures in children and care departments Related nursing 1. Lp and bone marrow aspiration 2. Liver biopsy 3. The correct principles of suctioning children and the possible side effects of suctioning 4. Taking care of catheters connected to the child (chest tube, Foley, vein catheter central and…) 5. Fever, ways to control fever, complications of fever in children	
The Fourth session	Determination of drug dose and serum therapy in children Nursing care before and after common surgeries	
The Fifth session	Basic resuscitation in children	
The Sixth session	advanced resuscitation in children	
The Seventh session	Nursing care of patients undergoing peritoneal dialysis Child care under mechanical ventilation How to work with mechanical ventilation Interpretation of ABG	
The Eighth session	Asthma, allergy in children and related nursing care	
The Nineth session	Nursing care in patients with congenital heart diseases	
The Tenth session	Diabetes in children and related points in nursing	
The Eleventh session	Nursing care in pediatric emergencies (poisoning, convulsions, respiratory problems, etc.)	
The Twelfth session	Triage of children	

The training content was confirmed by two professors at the Department of Pediatrics of Kerman University of Medical Sciences (a pediatrician and a pediatric nurse practitioner).

The participants had to participate in each session and study the materials for two weeks after the end of the intervention. The intervention was completed in four weeks. The researcher had full control over the participants at the beginning, middle, and end of the training course. To enhance the efficacy of learning, the lecturers used a combination of methods such as lectures, discussions, scenarios, films, slides, and photos. Moreover, all the participants could ask their questions during and after the online session. The researcher sent a reminder text message to the participants every day in order not to miss the online sessions and she checked their profiles to monitor their arrival and exit in the sky room system. The participants in the intervention group again completed the questionnaires in the Pors line system one month after the intervention. To prevent the exchange of information between the intervention and control groups, we asked the participants in the intervention group not to share the information with the participants in the control group until the end of the study.

## Control group

Nurses in the control group received no training in pediatric care. After arrangements with the system administrator, each nurse in the control group was given a username and password and completed the questionnaires before and one month after the intervention in the Porsline. The main researcher sent a reminder text message to them in order not to forget to complete questionnaires. After the study, the content of the e-learning program was provided to nurses in the control group.

## Instruments

The demographic information questionnaire and the Caring Assessment Questionnaire were used to collect data in this study.

## The demographic information questionnaire

This questionnaire included gender, age, marital status, educational level, work experience in the pediatric wards, shift work, position, and type of employment.

The Caring Assessment Questionnaire (Care-Q) was developed by Larson (1981) for assessing caring behaviors in the world and it is the most appropriate instrument for international comparison [28]. The Persian version of the Q-care includes 57 items with six subscales: being accessible (6 items), explains and facilitates (9 items), comforts (11 items), anticipates (5 items), trusting relationship” (18 items), monitors and follows through (8 items). The items were rated on a five-point Likert scale ranging from one (least important) to five (most important). The minimum score of this scale is 57, while the maximum score is 285. Pashaei (2014) validated the Persian version of this questionnaire using the back-translation method. The experts’ viewpoints were used to determine the content validity, while the test-retest method was applied to determine the reliability (r = 0.87) [29]. Content and face validities of the original version of the CARE-Q were previously established and test-retest reliability was assessed on 115 oncology nurses [28, 30].

### Data analysis

SPSS25 (SPSS Inc., Chicago, Ill., USA) was used to analyze the data. Descriptive statistics (percentage, frequency, mean and standard deviation) were applied to describe the demographic characteristics of the study participants. Chi-square and Fisher’s exact tests were also used to compare the intervention and control groups in terms of demographic variables. Based on the Kolmogorov-Smirnov test, independent samples t-test was used to compare the scores of Caring Assessment Questionnaire between the two groups at the pre-and post-intervention stages. Paired t-test was also used to compare the scores of Caring Assessment Questionnaire in each group at the pre-and post-intervention stages. Analysis of covariance was applied to control the impact of the pretest on the scores of Caring Behaviors Questionnaire. The significance level was set at P < 0.05.

## Results

The participants in this study were two groups of female nurses (n = 70) working in pediatric wards and pediatric intensive care units. Most of the nurses in the intervention (68.3%) and control groups (73.3%) were 20–30 years old. We found no statistically significant differences in demographic characteristics between the two groups (such as age, marital status, level of education, a nursing degree, employment, shift work, attendance in training courses, and work experience). In other words, the two groups were homogenous in terms of demographic variables (p < 0.05) (Table 2).

**Table 2:** The participants’ demographic characteristics in the intervention and control groups

Variable	Categories		Intervention (n = 30)		Control (n = 30)		Results
			Number	%	Number	%	
Age (years)*		20–30	22	62.8	27	73.3	χ^2^(2) = 4.496 P = 0.164
		31–40	4	11.42	6	20	
		41–50	9	25.7	2	6.7	
Marital status*		Single	14	40	14	40	χ^2^(1) = 0.052 P = 0.811
		Married	21	60	21	60	
Education*		Bachelor’s degree	25	71.4	24	68.75	χ^2^(1) = 0.123 P = 0.839
		Master’s degree	10	28.6	11	42.85	
Employment*		Official	16	45.71	15	42.85	χ^2^(2) = 0.798 P = 0.690
		Contractual	9	25.7	12	34.28	
		Plan-based	10	28.6	8	22.85	
Shift work**		Fixed	6	17.14	7	20	P = 1
		Rotating	29	82.85	28	80	
Participation in training courses		Yes	5	14.28	10	28.57	χ^2^(1) = 2.422 P = 0.236
		No	30	85.71	25	66.7	
Work experience in PICU (year)*		1>	12	34.28	34.28	16.7	χ^2^(2) = 5.602 P = 0.200
		1–5	12	34.28	14	40	
		< 5	11	31.42	9	26.7	

*Chi-square test and **Fisher’s exact test

The independent samples t-test indicated no significant differences in the mean scores of care behaviors between the intervention (256.61 ± 5.16) and control groups (257.52 ± 3.99) before the intervention (P = 0.23), but it showed a significant difference in the mean scores of caring behaviors between the intervention (275.69 ± 6.52 ) and control groups ( 254.21 ± 3.15 ) after the intervention (P = 0.001).

(Table 3). We found a significant difference in all dimensions of caring behaviors between the two groups after the intervention. Moreover, all of the subscale scores increased in the intervention group.

**Table 3:** Between- and within-group comparison of the Caring Assessment Questionnaire and its dimensions in the intervention and control groups

variables	Group	Before	after	p-value(within -groups)
Being accessible	Intervention Control	25.22 ± 0.79 24.45 ± 1.4	28.12 ± 0.15 23.89 ± 1.12	0.01 0.52
Between-group	p-value	0.42	0.01	
Explains and facilitates	Intervention Control	40.72 ± 0.14 40.89 ± 1.1	43.12 ± 1.32 41.14 ± 0.12	0.012 0.25
Between groups		0.45	0.02	
Comforts	Intervention Control	50.12 ± 0.13 51.75 ± 0.41	54.11 ± 0.51 50.55 ± 0.45	0.001 0.42
Between two groups		0. 55	0.47	
Anticipates	Intervention Control	21.20 ± 0.52 22.12 ± 0.41	24.12 ± 0.12 21.92 ± 0.32	0.01 0.52
Between two groups		0.47	0.01	
Trusting relationship	Intervention Control	84.01 ± 0.15 83.23 ± 0.23	88.12 ± 0.24 82.58 ± 0.14	0.001 0.23
Between two groups		0.27	0.001	
Monitors and follows through	Intervention Control	35.25 ± 1.23 34.92 ± 0.12	38.10 ± 0.19 34.13 ± 1.02	0.01 0.41
Between two groups		0.52	0.002	
Total of caring behaviors	Intervention Control	256.61 ± 5.16 257.52 ± 3.99	275.69 ± 6.52 254.21 ± 3.15	0.001 0.15
Between two groups		0.23	0.001	

## Discussion

We decided to determine the effect of an online training program on the caring behaviors of pediatric nurses.

The study results showed that online training programs improved nurses’ caring behaviors. The statistical results indicated a statistically significant difference in the mean scores of the caring behavior between the two study groups after the intervention, so online education of the principles of pediatric nursing improved caring behaviors in the intervention group, while we found no change in the control group.

Jalali et al. supported our results and showed that online training improved the knowledge and attitude of nurses working in neonatal intensive care units (22). They used offline electronic files, while we used online training and different groups were interested in the use of virtual learning due to its availability. The use of various teaching methods increased the quality of teaching.

[27]. Puksa and Janzen showed that the students experianced content containing complex cognitive concepts, experiential learning as in relational practice, and psychomotor skill mastery as better suited for online classroom delivery[31].

Boczkowska et al. (2018) showed that e-learning improved the care performance of emergency nurses. [32], but a study reported negative outcomes of distance learning that distance learning gives a person a specific set of knowledge that can be considered not only a plus but also a minus. Studying remotely, a person deprives himself of many positive “side effects” of academic education. For example, the process of taking notes of long lectures trains the speed of writing, develops mechanical memory, and teaches on the go to isolate the most important fragments from the flow of information. All these skills are very useful in everyday life, but distance learning does not [33].

A systematic review study comparing offline and online educational methods showed that online learning has advantages to enhance undergraduates’ knowledge and skills, therefore, can be considered as a potential method in undergraduate medical teaching [34]. Also, in the current study, all sessions were conducted online.

Some studies reported that the nurses and nursing students were more satisfied with using e-learning due to the following reasons: quality of content, the importance of social interactions, active learning, effectiveness and convenience of the technology, as well as the quality of support received, patient-centered approach, time-saving, and self-directed learning [35] [36].in a study, Nurses stressed the importance of authentic scenarios and practice of skills in the workplace [37]. Nurses found higher satisfaction with e-learning than with videotape.

In the current study, although the nurses received online training, they could interact with the teacher during the class and share their experiences in caring for the cases presented in each session. One of the strengths of this study was the availability of all recorded files from the beginning to the end of the intervention; the educational content was patient-centered and nurses learned the required educational content in a clinical setting so that they could remember the theoretical content with clinical experiences.

Nicoll found that e-continuing education was more beneficial than lecture courses; otherwise, nurses felt satisfied with both e-learning and traditional programs [38].

According to the progress made in science, technology, and communication, new teaching and learning methods can help nurses to improve their knowledge and skills in pediatric nursing care. Continuous and accessible learning is a basic requirement for acquiring clinical skills, and electronic learning allows nurses to access learning content at any time and place. According to the study results, the researcher believes that the use of virtual education as a complement to traditional methods can be a suitable approach for the retraining of nurses. As a result, time and costs will be saved.

## Limitations

This study had some limitations: a self-report questionnaire measured the effectiveness of an educational program in improving the caring behaviors of pediatric nurses. Assessment of caring behavior might have been affected by the participants’ bias inherent in the self-report questionnaire. Finally, data collection was conducted one month after the intervention. Future longer follow-ups (3–6) are recommended to have more accurate results, determine the long-term impact of training, and assess the effect of educational courses on nurses. Another limitation of this study was that the participants were selected from a single medical center, so the generalization of the results should be done with caution.

## Data Availability

The datasets used and/or analyzed during the current study are available from the corresponding author upon reasonable request.
